# ATP-sensitive potassium channel (K_ATP _channel) expression in the normal canine pancreas and in canine insulinomas

**DOI:** 10.1186/1746-6148-1-8

**Published:** 2005-11-02

**Authors:** Vicky R Donley, Erin K Hiskett, Aimee C Kidder, Thomas Schermerhorn

**Affiliations:** 1 Kansas State University, Department of Clinical Sciences, 1800 Denison Ave, Manhattan, KS 66506-5606, USA

## Abstract

**Background:**

Pancreatic beta cells express ATP-sensitive potassium (K_ATP_) channels that are needed for normal insulin secretion and are targets for drugs that modulate insulin secretion. The K_ATP _channel is composed of two subunits: a sulfonylurea receptor (SUR 1) and an inward rectifying potassium channel (Kir_6.2_). K_ATP _channel activity is influenced by the metabolic state of the cell and initiates the ionic events that precede insulin exocytosis. Although drugs that target the K_ATP _channel have the expected effects on insulin secretion in dogs, little is known about molecular aspects of this potassium channel. To learn more about canine beta cell K_ATP _channels, we studied K_ATP _channel expression by the normal canine pancreas and by insulin-secreting tumors of dogs.

**Results:**

Pancreatic tissue from normal dogs and tumor tissue from three dogs with histologically-confirmed insulinomas was examined for expression of K_ATP _channel subunits (SUR1 and Kir_6.2_) using RT-PCR. Normal canine pancreas expressed SUR1 and Kir_6.2 _subunits of the K_ATP _channel. The partial nucleotide sequences for SUR1 and Kir_6.2 _obtained from the normal pancreas showed a high degree of homology to published sequences for other mammalian species. SUR1 and Kir_6.2 _expression was observed in each of the three canine insulinomas examined. Comparison of short sequences from insulinomas with those obtained from normal pancreas did not reveal any mutations in either SUR1 or Kir_6.2 _in any of the insulinomas.

**Conclusion:**

Canine pancreatic K_ATP _channels have the same subunit composition as those found in the endocrine pancreases of humans, rats, and mice, suggesting that the canine channel is regulated in a similar fashion as in other species. SUR1 and Kir_6.2 _expression was found in the three insulinomas examined indicating that unregulated insulin secretion by these tumors does not result from failure to express one or both K_ATP _channel subunits.

## Background

The ATP-sensitive potassium (K_ATP_) channel is a crucial component of the pancreatic beta cell insulin secretion pathway and a target for drugs that modulate insulin secretion. In the endocrine cells of the pancreas, K_ATP _channel is composed of two subunits: a sulfonylurea receptor (SUR 1), which contains two adenine nucleotide binding domains [1], and an inward rectifying potassium channel (Kir_6.2_), which conducts potassium ions [2]. The K_ATP _channel is important for sensing the metabolic state of the pancreatic beta cell and initiating the changes in membrane potential that precede insulin exocytosis. In the presence of high concentrations of glucose, beta cell metabolism of the sugar increases ATP production. The resulting rise in the cellular ATP/ADP ratio causes closure of K_ATP _channels, which bind ATP and ADP at different sites [3]. The decrease in potassium conductance caused by channel closure depolarizes the cell membrane and initiates a sequence of ionic events that ultimately trigger insulin secretion [3].

Drugs that target pancreatic K_ATP _channels are used to modulate insulin secretion. Diazoxide and the sulfonylurea class of drugs are the most important examples of drugs that alter K_ATP _channel activity as their principle mechanism of action. These drugs have opposite actions on K_ATP _channel activity which is reflected by their effects on insulin secretion. Diazoxide, a potent K_ATP _channel opener, inhibits insulin secretion, even in the presence of glucose, by "locking" K_ATP _channels in the open configuration, which prevents cellular depolarization and prevents the ionic events that normally precede insulin exocytosis [4]. In contrast, the sulfonylurea drugs have the opposite effect on K_ATP _channel activity. These drugs cause K_ATP _channel closure, even in the absence of glucose, which leads to membrane depolarization and enhanced insulin secretion [5]. SUR 1 was discovered when research into the mechanism of action of the sulfonylurea drugs indicated that these drugs displayed high-affinity binding to a protein that modulated insulin secretion [6]. Diazoxide also binds to SUR 1, although at a different site than the sulfonylureas, since diazoxide does not prevent sulfonylurea binding [7,8]. Endogenous SUR1 ligands, such as alpha-endosulfine, may also regulate insulin secretion [9].

Although sulfonylurea drugs and diazoxide have the expected effects on insulin secretion from normal dogs [10-13], little information is known about the canine beta cell K_ATP _channel that is the molecular target for these drugs. Some but not all canine insulinomas respond to diazoxide [14], suggesting the possibility that mutations in the K_ATP _channel could be responsible for drug failure in some cases. Unregulated insulin secretion by the insulinoma could, in theory, result from any mutation of either SUR 1 or Kir6.2 that promotes K_ATP _channel closure or impairs normal metabolic regulation. Unfortunately, little has been reported about K_ATP _channel mutations in human insulin producing tumors and the authors are not aware of any reports examining the K_ATP _channel in canine insulinoma. However, naturally occurring mutations of SUR1 and Kir_6.2 _are known to cause a group of congenital hyperinsulinemic disorders of humans collectively known as persistent hypoglycemic hyperinsulinism of infancy (PHHI) [15]. About 40 separate mutations in SUR1 and three mutations in Kir_6.2 _have been described thus far [15,16]. The development of methods to study the canine K_ATP _channel would permit more detailed study of canine beta cell function and insulin secretion. Likewise, these methods could also be used to learn more about canine insulinoma, in particular the contribution of abnormal K_ATP _channel function to the unregulated insulin secretion that characterizes these tumors.

The purpose of this study was to learn more about K_ATP _channel expression in normal canine pancreas and in insulin-secreting endocrine tumors of the canine pancreas. The study goals were twofold: to develop RT-PCR methods and reagents to study K_ATP _channel expression in the normal canine pancreas and to screen a series of canine insulinomas to determine whether these tumors have normal K_ATP _channel expression.

## Results

### SUR 1 expression in normal canine pancreas

**Figure 1:**
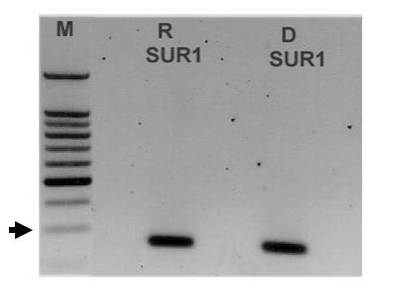
PCR primers designed against rat SUR1 amplified a single band of expected size (248 bp) from rat (R) and dog (D) pancreas. Sequence analysis confirmed the identity of each band as SUR 1. The size markers (M) are shown on the left. The arrow indicates the approximate location of the 300 bp marker.

**Figure 2:**
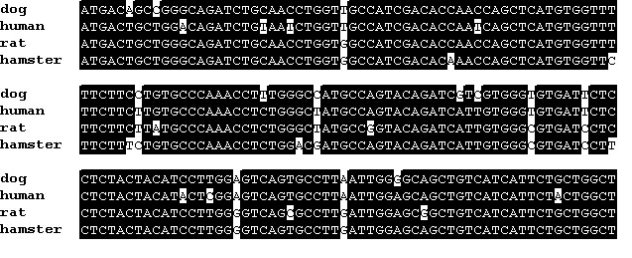
Canine SUR1 shares a high degree of nucleotide identity with SUR1 from other mammalian species. The alignment compares the partial sequence of canine SUR1 [GenBank:AY927669] with SUR1 from human [GenBank:AF087138], rat [GenBank:L40624], and hamster [GenBank:L40623]. Areas of sequence identity are shown in black and areas of non-identity are shown in white.

The results of RT-PCR for SUR1 expression in normal canine pancreas are shown in Figure 1. The primers, which were designed from the rat SUR1 nucleotide sequence, amplified a 248 bp product from rat and dog pancreases. Sequence analysis confirmed that the amplicon from rat pancreas was rat SUR1, indicating that the primers specifically amplified the desired product. The amplicon from normal dog pancreas was also determined to be SUR1 based on shared base identity with SUR1 from other species (Figure 2). The canine SUR1 sequence was verified by repeat sequencing and placed in GenBank [GenBank:AY927669]. The partial sequence of canine SUR 1 was found to have a high degree of nucleotide identity with corresponding SUR 1 sequences from humans (93%), rat (93%), and hamster (92%).

### Kir_6.2 _expression in normal canine pancreas

**Figure 3:**
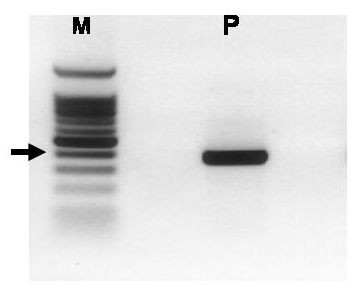
PCR primers designed against canine Kir_6.2 _amplified a single band of expected size (371 bp) from dog pancreas (P). Sequence analysis confirmed the identity of the band as Kir_6.2_. The size markers (M) are shown on the left. The arrow indicates the approximate location of the 400 bp marker.

**Figure 4:**
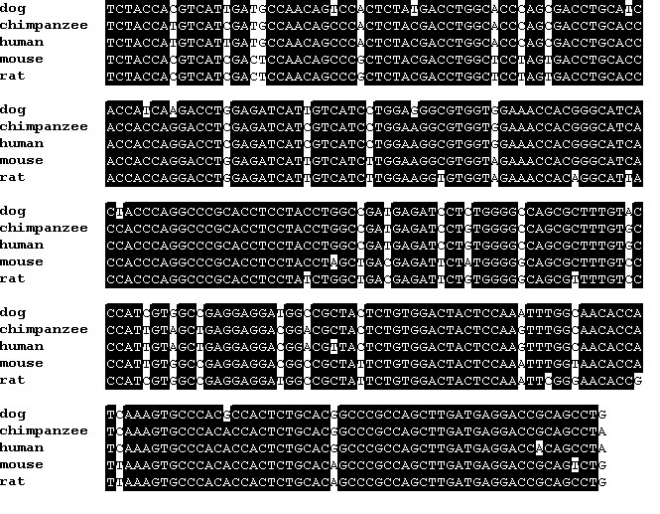
Canine Kir_6.2 _shares a high degree of nucleotide identity with Kir_6.2 _from other mammalian species. The alignment compares the partial sequence of canine Kir_6.2 _[GenBank:AY929390] with Kir_6.2_from chimpanzee [GenBank:XM521849], human [GenBank:BC040617], mouse [GenBank:NM-010602], and rat [GenBank:NM-031358] Areas of sequence identity are shown in black and areas of non-identity are shown in white.

Expression of Kir_6.2 _was detected in normal canine pancreas using primers designed against rat Kir_6.2 _[GenBank:D50581]. These primers amplified a product of expected size (approximately 167 bp) from rat and dog pancreases. Sequence analysis revealed that the PCR product from rat pancreas was 99% identical to the published sequence for Kir_6.2_, indicating that RT-PCR yielded a specific product. However, these primers did not produce clean, defined bands from canine pancreas (data not shown). To further clarify canine Kir_6.2 _expression, a second set of primers that were specific for canine Kir_6.2 _was designed using sequence information contained in the canine genome database [18]. These primers amplified a well-defined DNA band of expected size (371 bp) from normal canine pancreas (Figure 3). Sequence analysis showed that the canine product had 100% identity with a corresponding region of canine chromosome 21 [GenBank:AAEX01017445] contained in the canine genome database [18]. The canine pancreatic Kir_6.2 _sequence was verified by repeat sequencing and placed in GenBank [GenBank:AY929390]. The partial sequence obtained for canine Kir_6.2 _sequence shared extensive identity with Kir_6.2 _from other species (Figure 4). The canine Kir_6.2 _shares 95% identity with the predicted chimpanzee Kir_6.2 _but also has substantial identities with Kir_6.2 _from human (93%), mouse (90%), and rat (87%). The canine Kir_6.2 _was also compared to a predicted sequence for canine Kir_6.2 _[GenBank:XM-542519] that is available in GenBank. The 271 bp Kir_6.2_sequence from normal canine pancreas has 100% identity with the predicted nucleotide sequence of canine Kir_6.2_.

### K_ATP _channel subunit expression by canine insulinomas

**Figure 5:**
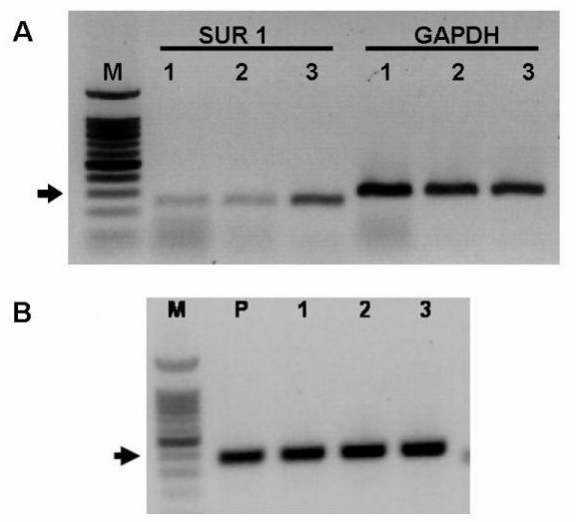
*Panel A *– PCR primers designed against rat SUR1 amplified a single band of the expected size (248 bp) from each of the three canine insulinomas examined (*lanes labeled SUR1 1–3*). Sequence analysis confirmed the identity of each band as SUR 1. GAPDH expression was used as a psoitive control (*lanes labeled GAPDH 1–3*). Semiquantitative analysis showed that SUR1 expression by insulinoma #3 was 3.5 and 2 fold greater than insulinomas #1 and #2, respectively (see text for explanation). Size markers (M) are shown on the left. The arrows indicate the approximate location of the 300 bp marker. *Panel B *– PCR primers designed against canine Kir_6.2 _amplified a single band of expected size (371 bp) from normal pancreas (P) and from each of the three canine insulinomas (labeled 1–3) examined. Sequence analysis confirmed the identity of each band as Kir_6.2_. Size markers (M) are shown on the left. The arrows indicate the approximate location of the 400 bp marker

Histologically-confirmed insulinomas from three dogs were analyzed for SUR1 and Kir_6.2 _expression. For each insulinoma, subunit expression was determined using the previously described rat SUR 1 and canine Kir_6.2 _primers. All three insulinomas were found to express the genes for SUR 1 and Kir_6.2 _(Figure 5). The identities of the PCR products obtained from each insulinoma were confirmed by nucleotide sequencing. Analysis of SUR1 and Kir_6.2 _sequences obtained from insulinomas did not reveal any mutations and were identical to the respective sequences obtained from normal canine pancreas. The intensity of SUR1 bands was noted to differ substantially among the insulinomas (Figure 5, panel A). Semiquantitative analysis comparing the OD of the SUR1 band with the OD of GAPDH band (the reference gene) from each insulinoma confirmed that SUR1 expression varied. Insulinoma #3 expressed SUR1 at a level that was 3.5-fold greater than insulinoma #1 and 2-fold greater than insulinoma #2. In contrast, expression levels of K_ir6.2 _were approximately equal in all three insulinomas (data not shown).

## Discussion

The results demonstrate that the SUR 1 and Kir_6.2 _subunits of the K_ATP _channel are expressed in the pancreas of normal dogs. The combination of SUR1 and Kir_6.2 _comprises the classical beta cell K_ATP _channel and expression of this subunit combination in the normal dog pancreas is consistent with previous reports showing that this same combination of subunits is expressed in the pancreatic beta cells by other species [2,19] and by insulin-secreting beta cell lines derived from the rat and mouse [2]. It is likely that the SUR1 and Kir_6.2 _detected in whole pancreatic samples represent islet specific expression since K_ATP _channel subunit expression has not been reported in the exocrine pancreas. SUR 1 and Kir_6.2 _expression is not specific to beta cells, however, and are also expressed in islet alpha cells, where they are involved in glucagon secretion [20,21]. As the K_ATP _channel subunits have similar function in all islet cells where they are expressed (e.g. sulfonylureas also stimulate glucagon secretion [21]) and since beta cells are the predominant endocrine cell in the canine islet, it may be concluded that the SUR1 and Kir_6.2 _detected in DNA isolated from whole pancreas reflects beta cell expression in the dog, although it is likely that other islet cells express the K_ATP _channel also.

Because the subunit composition is the same, we predict that the canine beta cell K_ATP _channel is subject to the same manner of metabolic regulation as the K_ATP _channel in other species, such as the rat, mouse, and human, which have an identical K_ATP _channel composition. The mechanisms of sulfonyluea and diazoxide on the pancreatic beta cell have not been studied at the molecular level in the dog but the results of this study predict that these pharmacologic agents will act via the K_ATP _channel to alter insulin secretion. Indeed, in vivo studies have shown the expected changes in serum insulin and glucose levels in dogs treated with sulfonylureas or diazoxide [10-13].

The RT-PCR results also showed that all three insulinomas examined expressed SUR 1 and Kir_6.2_. Thus, failure to express one or both subunits cannot account for the abnormal insulin secretion by these tumors. However, variation in SUR1 expression was noted among the insulinomas. In particular, expression in one tumor (insulinoma #3) was several fold greater than the other tumors. It is not known whether the expression level of one or both subunits plays a role in the abnormal insulin secretion by canine insulinomas. The expression of the K_ATP _channel by insulinomas is consistent with the hypothesized origin of these tumors from pancreatic beta cells or other pancreatic endocrine tissue. The islet origin of canine insulinomas is further supported by our findings that these tumors also express glucokinase and insulin but not amylase (V. Donley and T. Schermerhorn, unpublished observations).

The current results do not eliminate the possibility that the canine tumors express truncated versions of SUR1 or Kir_6.2_, such as those described for some humans with PHHI and unregulated insulin secretion [16,22,23]. Simple expression experiments do not provide information about functional aspects of the K_ATP _channel, however, and our experiments would not have been able to detect functional abnormalities of K_ATP _channels that might be present in the insulinomas. Many mutations do not affect gene expression but instead affect one or more of the steps beyond expression that are needed for proper channel function. Among the reported subunit mutations associated with K_ATP _channel dysfunction are mutations that cause impaired translation into functional proteins [16,22], abnormal protein trafficking [23], impaired protein folding [24], defective channel assembly [22,24], and altered nucleotide sensitivity and substrate binding [16]. Many of the known K_ATP _channel mutations leading to unregulated insulin secretion are associated with human PHHI. Most of these mutations affect SUR1 (there are over 40 reported human SUR1 mutations [15], while relatively few are Kir_6.2 _mutations [16,22,23]. Many of the mutations causing PHHI result in the production of K_ATP _channels that lack activity. In the absence of K_ATP _channel activity to couple metabolic activity and membrane potential, persistent calcium entry via voltage-sensitive calcium channels promotes insulin secretion that continues despite profound hypoglycemia. It is possible that mutations occurring in canine insulinomas might produce similar functional consequences. We screened canine insulinomas for mutations in SUR1 and Kir_6.2 _by comparing sequences obtained from insulinomas with those obtained from normal dogs. Using direct sequence comparison, no mutations were detected in the relatively short SUR1 or Kir_6.2 _sequences obtained from the insulinomas.

However, as the complete sequences of SUR1 and Kir_6.2 _from each insulinoma were not exhaustively screened for mutations, it is not possible to determine with any certainty whether or not K_ATP _channel mutations were responsible for the abnormal secretory function exhibited by these tumors. The partial DNA sequence of canine SUR1 obtained from these insulinomas is homologous to a region that spans exons 7 and 8 of human SUR1 which codes for the c-terminal end of the linker region between the 7^th ^and 8^th ^transmembrane regions as well as a portion of the 8^th ^transmembrane domain of the SUR1 protein. Several mutations in exon 8 have been found in humans with PHHI [16], suggesting that this region should be included when screening canine insulinomas for SUR1 mutations. However, the human SUR1 gene in humans is large, containing 39 exons [16]. Mutations causing hyperinsulinemia have been reported along the entire gene, including introns [15,16]. Assuming the canine gene is arranged similarly, mutation screening of SUR1 will not be readily performed until the complete nucleotide sequence of the normal canine gene is available. The same holds true for Kir_6.2_, although this gene is considerably smaller than SUR1 in the human and is likely to be similar in the dog.

## Conclusion

In conclusion, this study documents the expression of the K_ATP _channel subunits, SUR 1 and Kir_6.2_, in the normal canine pancreas. The partial sequences of canine SUR1 and Kir_6.2 _obtained are highly similar to those from other mammals, including humans, non-human primates, and rodents. K_ATP _channel subunit expression was detected in all three canine insulinomas examined, indicating that absence of subunit expression cannot account for the abnormal insulin secretion observed with these tumors. SUR 1 and Kir_6.2 _sequence comparisons with normal canine pancreas did not reveal any mutations within the sequences obtained from the canine insulinomas.

## Methods

### Animals

All normal pancreatic and insulinoma tissues were obtained from dogs submitted necropsy examination or from dogs that underwent surgery to remove an insulinoma. All pancreases used to determine K_ATP _channel expression in "normal" tissue was from dogs with no known pathology or that had non-pancreatic pathology. All dogs from which insulinoma tissue was obtained exhibited clinical signs suggestive of insulinoma and had a pancreatic mass at surgery. The diagnosis of insulinoma was confirmed by histological examination in each case. Rat pancreas served as control tissue for experiments unless otherwise indicated. Rat pancreatic tissue was obtained from normal Wistar rats that had been sacrificed for reasons unrelated to this study. Samples were immediately snap frozen in liquid nitrogen and maintained at -80 C until used for RNA extraction.

### PCR primers

Primers used in PCR reactions were designed against a rat sequence for SUR 1 [GenBank:L40624] and a mouse sequence for Kir_6.2 _[GenBank:D50581] [17]. A second set of Kir_6.2 _primers was designed in our laboratory using canine-specific sequence information recently made available through the canine genome project [18]. GAPDH expression was used as an internal control for each experiment. Primer pairs and their expected product size were SUR1 (sense 5'-GGAGCAATCCAGACCAAG AT-3'; antisense 5'-AGCCAGCAGAATGATGACAG-3', expected product 248 bp), rat Kir_6.2 _(sense 5'-TCCAACAGCCCGCTCTAC-3'; antisense 5'-GATGGGGACAAAACGCTG-3', expected product 167 bp); canine Kir_6.2 _(sense 5'-CCTACACCAGGTGGACATCC-3', antisense 5'-CAGGCTGCGGTCCTCATCAA-3', expected product 371 bp); GAPDH (sense 5'-ATCTTCCAGGAGCGAGAT-3'; antisense 5'-TGGTCATGAGTCCTTCCACGATA-3'; expected product 300 bp).

RNA isolation and reverse transcription-polymerase chain reaction (RT-PCR) RT-PCR was performed to determine the expression of SUR1 and Kir_6.2 _in canine pancreas and in a series of three canine insulinomas. GAPDH gene expression was used as an internal PCR control. To prepare RNA, frozen tissue samples were homogenized using a QIAshredder and RNA extracted in Trizol Reagent according to manufacturer's instructions (Qiagen RNeasy Mini Kit). cDNA synthesis was carried out using a mixture (19 μl) that included total RNA (5 μg), dNTP 10 mmol/L, random hexamers 100 ng/μl, RNasin 20 IU and reverse transcriptase (Superscript II RT, Invitrogen) 25 IU. The mixture was incubated for 50 min at 42 C according to manufacturer's instructions, after which the reaction was terminated by heating the reaction to 70 C for 15 min.

PCR amplification was carried out in 25 μL reactions that contained: 0.5 μL template cDNA, 1 μL of each specific oligonucleotide primer, 0.1 μL Invitrogen Platinum Taq DNA Polymerase, 2.5 μL 10× buffer, 0.75 μl MgCl_2_, 1 μL dNTPs and 18.95 μL distilled water. PCR cycles for amplification of SUR1 and Kir_6.2 _were as follows. SUR1: 94 C for 5 min; followed by 35 cycles of 94 C for 0.5 min, 59 C for 0.5 min, 72 C for 1.5 min and then 72 C for 30 min. Kir6.2: 94 C for 5 min; followed by 35 cycles of 94 C for 0.5 min, 58.6 C for 0.5 min, 72 C for 1.5 min; then 72 C for 30 min. Products from the PCR reaction were resolved by electrophoresis on a 1.2% agarose gel and detected with ethidium bromide staining and UV light. The identity of the PCR products was confirmed by sequence analysis performed using an automated ABI 3700 DNA Analyzer (Kansas State University, Department of Plant Pathology).

### Semiquantitative analysis of K_ATP _subunit expression in insulinomas

Optical densities (OD) of PCR bands were determined from gel images using ImageJ software (Rasband, W.S., ImageJ, U. S. National Institutes of Health, Bethesda, Maryland, USA, , 1997–2005). Semiquantitative analysis was performed by comparing the OD of the SUR1 and K_ir6.2 _bands to the OD of a reference gene (GAPDH) from the same insulinoma. The ratio of SUR1 (or K_ir6.2_) to GAPDH provides a semiquantitative basis for comparison of expression levels between different tumors.

## List of abbreviations

K_ATP _– ATP-sensitive potassium channel

Kir_6.2 _– inward rectifying potassium channel 6.2

PHHI – persistent hypoglycemic hyperinsulinism of infancy

OD – optical density

SUR 1 – sulfonylurea receptor 1

## Authors' contributions

VRD processed the tissues, performed the RT-PCR experiments, and prepared samples for sequencing. EKH participated in primer design, performed some of the RT-PCR experiments and participated in drafting the manuscript. ACK contributed important RT-PCR data. TS conceived and designed the study, drafted the manuscript, and performed sequence comparisons. All authors have read and approved the final manuscript.
